# Pseudotyped Viruses: A Useful Platform for Pre-Clinical Studies Conducted in a BSL-2 Laboratory Setting

**DOI:** 10.3390/biom15010135

**Published:** 2025-01-15

**Authors:** Sofiia N. Rizatdinova, Alina E. Ershova, Irina V. Astrakhantseva

**Affiliations:** Department of Immunobiology and Biomedicine, Sirius University of Science and Technology, 354349 Sirius, Krasnodarsky Krai, Russia; sophia_rizatdinova@mail.ru (S.N.R.); lina.ershova.99@gmail.com (A.E.E.)

**Keywords:** viral infection, in vitro model, pseudoviral particles, neutralizing antibodies, SARS-CoV-2

## Abstract

The study of pathogenic viruses has always posed significant biosafety challenges. In particular, the study of highly pathogenic viruses requires methods with low biological risk but relatively high sensitivity and convenience in detection. In recent years, pseudoviruses, which consist of a backbone of one virus and envelope proteins of another virus, have become one of the most widely used tools for exploring the mechanisms of viruses binding to cells, membrane fusion and viral entry, as well as for screening the libraries of antiviral substances, evaluating the potential of neutralizing monoclonal antibodies, developing neutralization tests, and therapeutic platforms. During the outbreak of severe acute respiratory syndrome coronavirus 2 (SARS-CoV-2), pseudotyped virus-based assays played a pivotal role in advancing our understanding of virus–cell interactions and the role of its proteins in disease pathogenesis. Such tools facilitated the search for potential therapeutic agents and accelerated epidemiological studies on post-infection and post-vaccination humoral immunity. This review focuses on the use of pseudoviruses as a model for large-scale applications to study enveloped viruses.

## 1. Introduction

Viruses are known to replicate only within living cells. In vitro models are frequently employed to examine the efficacy of antiviral agents and the functionality of the immune response, including vaccine-induced responses. In lieu of in vivo assays, the use of in vitro assays can facilitate the acquisition of comprehensive data on the ability of a virus to infect and persist in different cell types [1,2,3,4,5,6]. Moreover, this approach may prove invaluable in investigating the potency and efficacy of antiviral drugs and vaccines. The plaque assay and endpoint dilution assay offer the means of measuring the number of viral particles capable of replicating within a specific cell culture [7]. In a plaque assay, a serially diluted contaminated sample is applied to susceptible and permissive cells. The objective is to infect a cell with a single virion, which will replicate and propagate within the cell, resulting in the production of second-generation virions. These virions will then infect and kill surrounding cells, leading to the formation of a visible hole in the cell monolayer. The apparent concentration of the virus is often defined as plaque-forming units per volume of measure. This methodology is limited to viruses that induce cell lysis or death and, thus far, has remained the gold standard for quantifying the concentration of replication-competent lytic virions [8]. In the event that cells are not killed by the virus, but rather undergo morphological changes and form evaluable groups, focus-forming units are counted. In an endpoint dilution assay, the viral titer can be determined by calculating the tissue culture infectious dose (TCID) based on the presence of cytopathic effects (CPE). In the event that a virus does not result in the formation of CPE, an immunofluorescence assay is conducted in conjunction with the endpoint dilution assay, with the immunofluorescence being monitored instead of CPE. The most reproducible number is expressed as the dilution of the virus that produces pathological changes in half of the inoculated cells, which is referred to as the TCID50 [9].

Virus neutralization assays are typically conducted in conjunction with infectivity assays and are designed to detect antibodies (Abs) that reduce the infectivity of the virus or to assess the efficacy of specifically developed antiviral therapeutic agents [10,11]. There are numerous methods currently used in modern laboratory practice to evaluate the ability of neutralizing Abs (nAbs) or viral inhibitors to decline virus replication and propagation in the examined cells. One such method is the plaque reduction neutralization test (PRNT), which, like traditional approaches to virus research, is considered the gold standard for evaluating nAbs [12,13]. The fundamental configuration of the PRNT experiment entails the formation of virus–agent complexes, the introduction of a mixture to susceptible cells, incubation, and subsequent observation of emerging plaques [14]. An alternative assay has been developed to address the limitations of the PRNT. The focus-reduction neutralization test (FRNT) is analogous to the PRNT, employing a live virus strain and diluting the antibody or a serum of interest to varying concentrations before inoculation into a cell monolayer, with subsequent monitoring of the cytopathic effect (CPE) [15].

The study of highly pathogenic viruses is typically conducted in laboratories that adhere to rigorous biosafety standards, predominantly at levels 3 and 4, as recommended [16].

This obstacle limits the number of organizations able to work with these agents, thereby impeding the pace of academic research and therapeutics development in the field of virology. In light of these limitations, it is important to pursue alternative approaches that can overcome these constraints.

The pseudovirus neutralization test employs packaging-convenient and replication-defective pseudotyped viruses (pseudoviral particles, PVPs) that display the target surface protein of the virus of interest. A comparison of pseudovirus assays with those conducted using live viruses reveals a high degree of convergence in the resulting data [17,18]. Moreover, pseudotyped virus-based assays can be conducted in biosafety level 2 (BSL-2) laboratory facilities. This approach enables both qualitative and quantitative evaluation and is suitable for large-scale detection [19].

## 2. Types of Pseudoviral Particles

Pseudoviruses are recombinant viral particles whose core skeleton is derived from one type of virus, whereas their surface protein is derived from another [20,21,22]. One of the most noteworthy characteristics of PVPs is their capacity to infect susceptible host cells with a single round of replication. The replication defectivity of PVPs is a direct consequence of alterations and modifications to their genes. Consequently, PVPs offer significant advantages in the study of cellular tropism, receptor recognition, virus inhibition, and the development and evaluation of vaccines used in animal models and clinical trials [23,24,25].

In general, the production of viral vectors is conducted through the co-transfection of permissive cells with multiple plasmids encoding a viral genome. The resulting particles are typically non-propagating due to the absence of essential genes required for reproduction. They are pseudotyped to transduce a specific type of cell [26].

### 2.1. Recombinant Lentiviruses

Over the past three decades, numerous packaging systems have been investigated with the objective of developing envelope-pseudotyped viruses. One of the earliest and most widely utilized options is the lentiviral vector packaging system [27,28]. This system is based on lentiviral vectors, which are mainly derived from species including Human immunodeficiency virus 1, Human immunodeficiency virus 2, Feline immunodeficiency virus, Simian immunodeficiency virus, and Equine infectious anaemia virus.

Natural lentiviral particles possess enveloped, spherical or pleomorphic capsids with a diameter ranging from 80 to 120 nm. They contain a positive, single-stranded RNA genome that is reverse-transcribed into DNA and integrated into the host cell genome, exhibiting a preference for transcriptionally active sites. In the case of human immunodeficiency virus type 1 (HIV-1), the productive entry requires a delayed endocytosis step [29].

**Figure 1:**
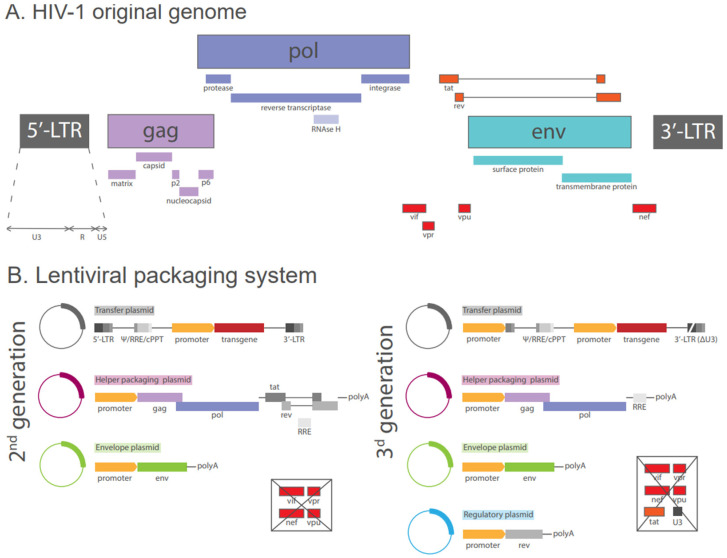
Schemes of original HIV genome and lentiviral packaging systems based on it. (**A**) Schematic representation of HIV-1 original genome: LTR—long terminal repeat essential for viral transcription, reverse transcription, and integration; *gag*—gene encoding for proteins p24 (formation of the capsid), p17 (formation of the inner membrane layer), p7 (formation of the nucleoprotein/RNA complex), p6 (virus particle release); *pol—*gene encoding a set of enzymes including p10 (proteolytic cleavage of precursor proteins), p51 (transcription of HIV-1 RNA in proviral DNA), p15 (degradation of viral RNA in the viral RNA/DNA replication complex), p32 (integration of proviral DNA into the host genome); *env*—gene encoding for surface glycoprotein gp120 (attachment to the target cell), transmembrane protein gp41 (anchorage of gp120, fusion with the target cell); *tat*—gene encoding for regulatory protein p14 (activator of transcription of viral genes); *rev*—gene encoding for RNA splicing-regulator protein p19 (regulation of the export of non-spliced and partially spliced viral mRNA); *vif*—gene encoding for viral infectivity protein p23 ; *vpr*—gene encoding for virus protein r, or p15 (component of virus particles, interaction with p6); *vpu*—gene encoding for virus protein unique, or p16 (efficient virus particle release, control of CD4 degradation); *nef—*gene encoding for negative regulating factor, or p27 (influence on HIV replication, enhancement of infectivity, downregulation of CD4 on target cells). (**B**) Recombinant packaging systems comprising second and third generations of lentiviral vectors: Ψ—psi viral packaging signal sequence (packaging and delivering of transgene mRNA); RRE—cis-acting Rev response element (export from the nucleus to the cytoplasm of viral transcripts); cPPT—central polypurine tract (improvement of the vector integration and transduction efficiency); polyA—(promotion of transcript longevity); *env—*gene coding for glycoprotein of the virus of interest (replacement of the native lentiviral Env glycoprotein, providing vectors to transduce a particular set of cells); ΔU3—self-inactivating 3′LTR (viral transcription, reverse transcription and integration; contains a safety measure to prevent viral replication).

The original HIV-1 genome is approximately 9.7 kilobases in length. As illustrated in Figure 1A, the proviral genome is flanked by two long terminal repeats (LTRs) comprising the U3, R, and U5 regions and encompasses three structural genes. The *gag* gene encodes proteins that are integral to the outer core membrane (MA, p17), the capsid protein (CA, p24), the nucleocapsid (NC, p7), and the nucleic acid-stabilizing protein. The *pol* gene encodes several proteins, including protease (PR, p12), reverse transcriptase (RT, p51), RNase H (p15), and integrase (IN, p32). The *env* gene encodes two envelope glycoproteins, namely the surface protein (gp120, SU) and the transmembrane protein (gp41, TM), which define the virus’s tropism and enable its entry into host cells [30]. HIV-1 employs the CD4 receptor and either the C-C chemokine receptor type 5 (CCR5) or the C-X-C chemokine receptor type 4 (CXCR4) co-receptors located on the host cell membrane to initiate an entry process [31].

To mitigate the risk and to increase safety in research contexts, three generations of lentiviral packaging systems were developed. The initial generation of HIV-1-derived packaging systems was constructed as a three-plasmid configuration. The transfer plasmid contained the transfer vector with the Ψ packaging signal, the cis-acting sequences necessary for reverse transcription and integration, and an internal promoter for the regulation of transgene expression. A reporter gene was used as a transgene to detect the entry of the pseudovirus into the cell. Usually, reporters coding the luciferase or fluorescent protein genes were used. A helper packaging plasmid represented a recombinant HIV-1 genome with a heterologous promoter for the expression of HIV-1 Gag/Pol, but lacking the *env* gene. The envelope plasmid included the *env* gene, which delivered the lentiviral envelope or the desired pseudotyping proteins, thus providing receptor binding and membrane fusion with the target cell. However, the first-generation system is no longer employed in current practice due to safety concerns. Early replication-defective HIV vectors could incorporate the HIV *env* gene and exhibit a substantial risk of generating wild-type HIV by recombination of the constructs employed for vector production [32].

Second-generation vectors were further improved with regard to safety considerations. The HIV-1 genome was still edited and distributed across the three plasmids. The transfer plasmid encoded for a transgene, and the packaging plasmid contained only essential *gag*, *pol*, *rev*, and *tat* genes, thus lacking genes for envelope proteins, Vif, Vpr, Vpu, and Nef accessory proteins. The envelope plasmid encoded the genes necessary for the production of envelope proteins (Figure 1B).

In third-generation vectors, the original helper packaging plasmid was divided into two expression plasmids: one encoding Rev and one encoding Gag and Pol. The incorporation of a discrete rev plasmid additionally diminished the likelihood of recombination. Moreover, the long terminal repeats (LTRs) were modified by the removal of the enhancer and promoter region U3 in both instances. Additionally, a constitutively active heterologous promoter was introduced upstream of the vector transcript in the 5′ LTR, thereby replacing Tat as a transcription activator. This eliminated the need for the *tat* gene and enhanced biosafety considerations for the platform (Figure 1B). Although the third-generation system was considered the safest, the viral yield was found to be significantly lower than that of the second-generation system [33].

Episomal integrase-deficient lentiviral vectors represent a modification of the classical third-generation lentiviral packaging systems. These vectors lack the machinery necessary for genome integration, resulting in a weak integration capability and transient expression in the cell [34].

Similarly, other lentiviral-based platforms are capable of efficiently delivering genes into a multitude of cell types, integrating an insert into the host cell genome, and generating long-term, stable transgene expression. The potential of non-human lentiviral vectors for gene therapy was demonstrated in the treatment of a range of diseases, including glycogen storage disease type Ia, glaucoma, and neovascular age-related macular degeneration [35,36].

In general, classical lentiviral vectors are distinguished by their capacity for the permanent integration of vector DNA, which results in the sustained, long-term expression of the product of interest. They provide the potential for achieving high viral titers, which in turn allow for the attainment of high transduction efficiency in both cultured cells and live animals. From a practical standpoint, they demonstrated superior packaging capacity compared to other vectors, such as adeno-associated vectors, and were described as packaging systems with broad tropism. The transduction of a wide range of cells and tissues in lentiviral vectors is typically dependent on the vesicular stomatitis virus G protein (VSV-G), which is encoded in the envelope plasmid. From the perspective of experimental analysis, as lentiviruses undergo the lysogenic cycle, resulting in the release of the virus into the medium without cell lysis, flow cytometry can be used to determine the functional viral titers, which is a more accurate method compared to those based on physical titers. In regard to gene delivery for clinical applications, the absence of interference from pre-existing viral immunity represents another advantage of lentiviral vectors. Nevertheless, there are several constraints associated with the utilization of lentiviral packaging systems. These include the potential for the generation of replication-competent viruses and the risk of insertional mutagenesis and subsequent genotoxicity due to the integration-associated induction of aberrant splicing of cellular transcripts [37].

### 2.2. Recombinant Rhabdoviruses

**Figure 2:**
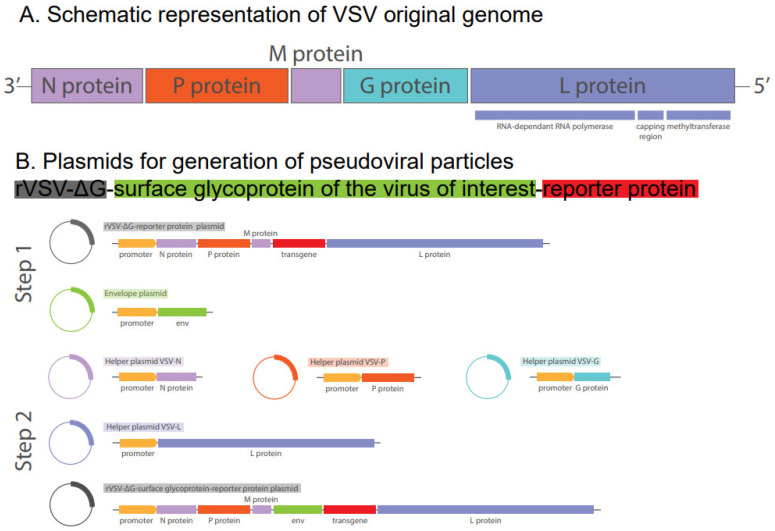
Schemes of the original VSV genome and the packaging system based on it. (**A**) Schematic representation of a single-stranded negative-sense RNA genome consisting of five primary genes coding for: N protein—nucleoprotein; P protein—phosphoprotein; M protein—matrix protein; G protein—glycoprotein; L protein [38]. (**B**) Recombinant packaging system. Step 1: Generation of rVSV-ΔG-envelope-transgene, where the envelope encodes the surface glycoprotein of the virus of interest and the transgene encodes the reporter protein. Step 2: Co-transfection with the obtained plasmid and VSV-system helper plasmids, resulting in rVSV-ΔG-surface glycoprotein of the virus of interest-reporter protein production [47,48].

Since the 1990s, recombinant rhabdovirus recovery expression systems have been developed, with a particular focus on rabies virus, vesicular stomatitis virus (VSV), and Maraba virus [38]. VSV vectors are derived from the species *Vesiculovirus indiana*, which belongs to the family *Rhabdoviridae* and the genus *Vesiculovirus.* VSV are enveloped viruses with a bullet-shaped capsid, measuring between 70 and 200 nm in diameter. They contain a negative-sense, single-stranded RNA genome that encodes for several viral proteins, including nucleoprotein (N), matrix protein (M), glycoprotein (G protein), viral polymerase (protein L), and phosphoprotein (P) (Figure 2A) [38]. The G protein is responsible for receptor recognition, cell entry and viral fusion. It employs low-density lipoprotein receptors and gains entry into host cells via a clathrin-mediated endocytic pathway, subsequently undergoing fusion of the viral envelope with the endosomal membrane, facilitated by the G protein [39,40]. Following the release of the virion core into the host cell cytoplasm, the M protein undergoes a dissociation from the N protein. Transcription and replication are driven by the viral polymerase complex, composed of the L and P proteins, and the entire process is considered typical of single-stranded, negative-sense RNA viruses. The newly generated genomes serve as templates for secondary transcription of viral genes, which represents the primary amplification step of the replication cycle. Upon condensation of the nucleocapsids through interactions with the M protein, budding of nascent virions occurs from the plasma membrane at sites that are enriched in the G protein [41,42].

First studies have demonstrated that the coinfection of cells with VSV and a second virus results in the formation of a pseudotyped virus that carries the VSV core and envelope proteins derived from the second virus [43]. VSV assembly occurs at the membrane, whereby virions bud from the cell surface. Furthermore, it has been demonstrated that VSV virions are not selective with regard to the proteins that can be incorporated into the viral envelope at the time of budding [44].

To develop the pseudoviral systems reverse genetics methods were employed [45,46]. In order to achieve this objective, plasmids that code for an analogue of the VSV antigenomic RNA were utilized, incorporating a ribozyme sequence derived from hepatitis delta virus for the purpose of obtaining precise 3′ ends. The N, P, and L proteins were provided in trans from so-called “helper” plasmids. The expression was driven by the T7 promoter.

It is important to note that VSV is capable of functioning in the absence of G protein. Consequently, the coding gene for G protein can be replaced with the gene encoding reporter proteins. Recombinant VSV particles with deleted G proteins (rVSV-ΔG) undergo a single cycle of infection, and their cell tropism and entry properties are typically dependent on the addition of a heterologous glycoprotein (Figure 2B). It is important to note, however, that the deletion of G protein genes has been observed to result in a reduction in PVP production [49]. The VSV packaging system has the capacity to accommodate approximately 6 kb of foreign sequences. It is distinguished by a high viral titer, a lack of reassortment, an inability of the viral RNA to integrate into the host genome, an ability to be efficiently pseudotyped with envelope glycoproteins derived from heterologous viruses, a low seroprevalence in the general human population, and the induction of both humoral and cellular immune responses [50]. It is important to note, however, that there are some limitations associated with VSV production. These include limited cargo space and technical complexity, due to the fact that VSV production involves multiple steps [51].

To sustain the elevated level of PVP production, the rVSV-ΔG bearing G protein (rVSV-ΔG-G) is utilized. The fundamental protocol for the production of VSV pseudo-types entails the transfection of a cell line with a viral surface glycoprotein of interest, followed by inoculation with rVSV-ΔG-G. Subsequently, the pseudotypes are harvested after a designated period in the presence of nAbs specific to the VSV G protein [52]. Moreover, it has been demonstrated that the efficacy of the protocol may be enhanced by utilizing a cell line that stably expresses the viral surface protein under examination. Fujioka et al. have demonstrated that the utilization of a cell line that stably expresses a fluorescently tagged S protein of SARS-CoV-2 on its surface results in the production of a higher amount of PVPs harbouring S protein in comparison to a cell line that transiently expresses such a protein. The productivity of PVPs may also be considerably influenced by the cell-producing line [53].

### 2.3. Other Recombinant Retroviruses

Enveloped pleomorphic, but roughly spherical, with a diameter of 80 to 120 nm single-stranded positive-sense RNA gammaretroviruses, often referred to as retroviruses, possess a packaging capacity of around 8 kb of foreign sequences [54]. In contrast to lentiviruses, which also belong to the family *Retroviridae*, they are unable to transduce non-dividing cells. The underlying reason for this phenomenon is nuclear impermeability, which prevents virions from transducing cells unless they are undergoing division, at which point the nuclear envelope breaks apart. Examples of gammaretroviral vectors include the packaging systems of the murine leukaemia virus (MLV) and feline leukaemia virus (FLV), which are based on viruses belonging to the family *Retroviridae*, genus *Gammaretrovirus* [55].

MLV is a C-type retrovirus with a simple genome that lacks inhibitory elements, requires only one splicing event (to produce the Env mRNA), and does not encode for accessory proteins [56]. The genome of MLV contains four genes that are common to all retroviruses: *gag*, *pro*, *pol*, and *env*. The *gag* coding region is responsible for the production of the structural proteins. The *pro* and *pol* genes encode the protease, reverse transcriptase, and integrase enzymes. Finally, the *env* gene is responsible for the production of the envelope glycoprotein, which binds to the target cell receptor and mediates virus–cell membrane fusion [57].

In order to utilize MLV as transfer vectors, it has been rendered replication-defective. The modifications include the development of a split packaging design, whereby genes encoding all essential viral components are provided on separate plasmids for vector production. Consequently, the transfer vector is devoid of all protein-coding reading frames, thereby allowing the inclusion of a recombinant expression cassette [58].

The production of MLV vectors is analogous to that of HIV–lentiviral vectors and entails the transient transfection of envelope, packaging, and transfer plasmids into HEK293F cells [59]. Where heterologous envelope protein encoding by envelope plasmid, packaging plasmid encoded genes *gag* and *pol* and transfer plasmid used to encode gene of interest flanked by the viral long translation regions (LTRs). A major deletion of the viral promoter sequences within the U3 region of the LTR abolishes transcriptional activities of integrated vectors, rendering the vector replication incompetent. In lieu of a viral LTR-driven promoter, an internal heterologous promoter is utilized to express the vector-encoded transgene. This promoter is derived from a variety of sources, including CMV, spleen focus-forming virus (SFFV), elongation factor 1 alpha (EF1α), and others [58].

The original MLV employs both non-endocytic and endocytic pathways for cellular entry, with initial binding to the cationic amino acid transporter MCAT-1 [60]. Recombinant MLVs are designed to be infectious but non-propagating and are employed for the gene therapy of monogenic diseases such as Wiskott–Aldrich syndrome and junctional epidermolysis bullosa [61]. Furthermore, these vectors are employed in pseudotyped-virus-based assays for the investigation of the tropism and host entry pathway characteristics of highly pathogenic viruses [62]. They share with lentiviral vectors such features as permanent integration of vector DNA into the host genome, a large cargo space, high-level and stable transgene expression, but were demonstrated to have a moderate viral titer and safety issues due to the risk of insertional mutagenesis.

In order to address the issue of a PVP yield that is insufficient for clinical-grade vector production in adherent packaging cell lines, a new suspension packaging cell line has been developed in recent years. This enables the production of MLV-based PVPs in bioreactors [63].

## 3. Pseudotyped Virus-Based Assay Application for Study COVID-19

### 3.1. Study of Cellular Susceptibility and the Mechanism of Viral Entry

PVP has been employed extensively in the study of the interaction between the virus and host cells. This was employed to examine the interaction between SARS-CoV and SARS-CoV-2 and host cellular receptors [52,64]. Yang and colleagues utilized wild-type, XBB.1.61, or B.1.617.2 lentiviral-based pseudotyped SARS-CoV-2 particles with reporter genes of GFP (green fluorescent protein) and luciferase to demonstrate that PVPs can significantly infect hepatic Huh-7 and HepG2 cells. However, the simultaneous knockout of ACE2 and asialoglycoprotein receptor 1 (ASGR1) prevented SARS-CoV-2 pseudovirus infection, suggesting that ASGR1 promotes SARS-CoV-2 entry into human hepatocytes [65]. Moreover, the presence of the cholesterol uptake receptor Niemann-Pick type C1-like 1, an endosomal membrane protein that regulates intracellular cholesterol trafficking and exhibits high expression levels in several organs, including the kidneys, has been demonstrated to facilitate SARS-CoV-2 entry in vitro using a PVP-based assay [66]. Melano et al. demonstrated the relationship between Wnt3a, a powerful activator of the Wnt/β-catenin signalling pathway, and facilitated ACE2-mediated virus infection. Using lentiviral-based PVPs with a luciferase reporter gene, it was demonstrated that SARS-CoV-2 pseudotyped virus infection with Wuhan, Delta and Omicron variants activates the Wnt/β-catenin signalling pathway, which subsequently stimulates ACE2 transcription [67]. Pellegrini et al. investigated the neurotropic potential of SARS-CoV-2 by challenging human pluripotent stem cell-derived brain organoids with lentiviral-based pseudotyped particles expressing GFP reporter gene. It was found that SARS-CoV-2 infection results in a damage to the choroid plexus epithelium, which subsequently leads to fluid leakage across the blood-cerebrospinal fluid barrier [68]. It has been demonstrated that GRP78 acts as a receptor for SARS-CoV-2 spike protein to mediate ACE2-independent virus entry into monocytes which express low or no ACE2 [69]. Furthermore, the utilization of the pseudovirus-based surrogate test in BSL-2 facilities has demonstrated that a plasma membrane and an appropriate protease are sufficient to facilitate SARS-CoV-2 pseudovirus fusion and the subsequent subcellular location after the entry. This process is likely dependent on the differential activity of airway, cell surface, and endosomal proteases, yet all of them are capable of supporting infection [70].

As the SARS-CoV-2 pandemic progresses, the receptor binding capacity of newly emerging variants of the virus is being investigated using PVP-based assays. The design of PVP carrying the S protein from different viral variants, and in some cases, carrying specific amino acid mutations, has enabled several groups to study the entry efficacy of different SARS-CoV-2 variants and to investigate the evolutionary path of the virus [71,72]. Furthermore, the diverse host factors that may impact viral entry can be investigated through the use of PVP assays. Recently, it was demonstrated that SH3 domain-binding protein 4 regulates the entry of PVP-carrying S protein from the Wuhan, Delta, and Omicron BA.2 variants in an integrin- and clathrin-dependent manner [73].

### 3.2. Entry Inhibitors Screening, Identification and Evaluation

Since PVP-based assays provide a great deal of insight into the entry mechanism used by SARS-CoV-2, the same platforms could be used to screen for potential entry inhibitors or to preclinically evaluate the selected one. The PVP-based technology is employed as a conformational assay for viral entry inhibitors following in silico analysis of the candidate and subsequent screening methods [74,75].

Palla and colleagues devised and synthesized a class of pyrrolidinones with the objective of reversibly inhibiting of transmembrane protease serine 2 (TMPRSS2) and furin. The use of SARS-CoV-2 PVPs enabled the confirmation of the blocking effect of the most potent inhibitor on virus ACE2-dependent entry, thereby demonstrating the potential of the compound for further preclinical investigation [76]. In a study by Kazakova and colleagues, the antiviral activity of a series of triterpene A-ring hydroxymethylene amino derivatives was investigated. The compounds were evaluated for their potential to inhibit SARS-CoV-2 PVPs, and they demonstrated notable inhibitory activity [77]. Mei et al. proposed that a dual blockade of conservative host receptors, ACE2 and neuropilin-1, would constitute an advantageous pan-inhibition strategy. By employing structure-based virtual screening, the researchers were able to identify a dual-targeting peptide, AP-1, which demonstrated remarkable inhibitory activity against SARS-CoV-2 VOCs in a pseudovirus infectivity assay [78]. Razi et al. employed systematic evolution of ligands by exponential enrichment (SELEX) to identify fluoro-arabino nucleic acid aptamer R8-9, which was found to interfere with the interaction between the SARS-CoV-2 RBD and ACE2. Moreover, it was demonstrated that the selected aptamer inhibits the uptake of spike-bearing VSV-based PVPs by cells [79]. Other researchers demonstrated that the ACE2 receptor facilitated, but was not a prerequisite for, SARS-CoV-2 membrane fusion. In order to test the hypothesis that DNA-lipid tethering could serve as a synthetic attachment factor in place of ACE2, experiments were conducted using two viral particle types: HIV-based pseudoviruses and virus-like particles created by co-expressing S, E, M, and N proteins [80]. The possibility of using the ACE2 peptide for prophilaxis of SARS-CoV-2 infection was also evaluated [81,82].

In addition, the wide range of naturally derived plant-based virus inhibitors used in traditional medicine have been evaluated using the PVP-based system [83,84,85,86]. PVP pseudotyped with S protein from different viral variants, including recently emerging ones, gave the scientific community an insight into the breadth and depth of inhibitor efficacy.

Furthermore, the PVP-based platforms facilitate the investigation of the efficacy of entry inhibitors for co-infections of different viruses, including co-infections with SARS-CoV-2 and influenza. Additionally, the analysis can be extended to encompass different variants of both viruses, utilizing particles pseudotyped with a range of variants of the SARS-CoV-2 S protein and influenza A haemagglutinin [87].

Moreover, K18-hACE2 mice infected with SARS-CoV-2 spike pseudovirus were employed as an in vivo model for the assessment of the efficacy of TMPRSS2 and cathepsin L inhibitors [88]. The scientists established a panel of nanocarriers coupled with anti-hACE2 Abs, which were loaded with TMPRSS2 or/and cathepsin L inhibitors (Camostat or/and E-64d, respectively). In an in vitro model, it was demonstrated that a combination of TMPRSS2 and cathepsin L inhibitors more effectively inhibited the entry of SARS-CoV-2 spike pseudovirus into HEK-hACE2 cells. Treatment of mice infected with luciferase-labelled SARS-CoV-2 spike pseudovirus with that combination of protease inhibitors resulted in significantly reduced luminescence in the infected lung area compared to uninfected controls. Moreover, the implementation of a combination of TMPRSS2 and cathepsin L inhibitors not only hindered infection, as evidenced by a decrease in or absence of luminescence signal from the lung area on Day 2, but also prevented major organ lesions.

### 3.3. Therapeutic Antibodies Evaluation

A pseudoviral neutralization assay was incorporated into the pipeline for in vitro evaluation of monoclonal antibodies (mAbs) against wild-type SARS-CoV-2 and its variants. Wang et al. constructed an immunoglobulin-like bispecific antibody, designated BI31, by fusing two parental mAbs that target the S protein RBD. In the PVP test, BI31 demonstrated neutralizing effects against SARS-CoV-2, SARS-CoV, and Middle East respiratory syndrome coronavirus, suggesting it has the potential to become a broad-spectrum therapeutic antibody [89]. The pseudoviral neutralization assays facilitate the point the virus variants that are targeted by mAbs. Li Ko et al. identified a potent SARS-CoV-2 antibody, designated SKAI-DS84, through the use of phage display technology. The authors subsequently confirmed the antibody’s capacity to neutralize wild-type, B.1.617.2, and B.1.1.529 subvariants of SARS-CoV-2 using a lentivirus-based nano-luciferase expressing SARS-CoV-2 pseudoviruses [90]. Gao et al. generated a novel monospecific tetravalent IgG1-(scFv)2 version of mAb 553-15 by fusing two forms of 553-15-derived single-chain variable fragments to the C-terminus of the human immunoglobulin G1 Fc fragment. In comparison to the original monoclonal antibody, the enhanced neutralization observed in PVPs assays against VOCs is noteworthy [91]. AZD3152 mAb neutralized a broad panel of pseudovirus variants, including the once dominant Omicron variant JN.1 but reduced potency against XBB subvariants containing F456L [92]. The assistance of pseudoviral-based assays, which utilized the SARS-CoV-2 S proteins from Alpha, Beta, Delta and Omicron (B.1.1.529, BA.2, XBB, and XBB.1.5) variants, enabled the study of the nanobodies which had previously demonstrated neutralizing activities against the Wuhan strain. The nanobodies were obtained through llama immunization with recombinant SARS-CoV-2 S-2P and RBD proteins, subsequently leading to the identification of specific antibody sequences. An analysis focused on 29 nanobodies, exhibiting notably higher levels of expression. However, only 15 of those nanobodies have been shown to possess the capacity to neutralize SARS-CoV-2 pseudovirus infection in vitro, in a model derived from the Wuhan variant. Moreover, the cocktail of nanobodies that was able to neutralize the new variants of the virus was also identified [93].

### 3.4. Pseudovirus Neutralization Assay for Detection Neutralizing Antibody

Since the onset of the SARS-CoV-2 pandemic, there has been a continuous development of validated pseudovirus-based assays with PVPs bearing the S protein of different SARS-CoV-2 variants [94,95,96]. They were employed extensively in clinical trials designed to assess post-vaccination humoral immunity which allow to study how the effectiveness of post-vaccination immunity changes against different variants of the virus, to evaluate and compare the effectiveness of various vaccines and the duration of post-vaccination immunity [97,98,99,100,101]. The use of PVPs carrying the spike protein of distinct virus variants allowed to evaluate the cross-reactivity of these variants and predict the effectiveness of post-vaccination immunity [102,103,104].

Furthermore, PVPs were employed in regional studies investigating herd immunity to SARS-CoV-2, including protective immunity against the recently emerged variants [66,105]. Pseudovirus tests were used in preclinical trials of new vaccine variants to assess efficacy against both circulating and “historical” variants [106,107]. It was shown that antibodies produced in response to infection with different SARS-CoV-2 variants were characterized by different cross-reactivity to other viral variants. Thus, SARS-CoV-2 Omicron, BA.4/5 infection triggered highly cross-reactive functional antibodies compared to BA.1 strain [108]. Using the panel of pseudoviruses carrying artificially mutated S protein it was revealed that the strongest escape mutations are located in the RBD at sites 357, 420, 440, 456 and 473, but the mutations that significantly affected ACE2 binding are located outside RBD of SARS-CoV-2 [109].

The use of pseudotyped viruses in animal research revealed the existence of five distinct serotypes. For that purpose, VSV-ΔG-G-GFP pseudotyped viruses of 23 corresponding SARS-CoV-2 variants were employed in a virus neutralization assay to assess the cross-neutralization capacity of serially diluted antisera obtained from mice immunized with mRNA vaccines based on the RBD of 23 SARS-CoV-2 variants [110]. Pseudotyped lentiviral viruses bearing the S protein of the SARS-CoV-2 ancestral strain and VOCs (B.1 lineage, Alpha, Beta, Gamma, Delta, and Omicron BA.1) were employed to analyse cat serum samples in the veterinary study of SARS-CoV-2 prevalence and variant surveillance among cats in the USA [111].

The results of PVP neutralization tests demonstrated that a key deletion, designated DelS31, within the S protein of JN.1 is responsible for the virus’s capacity to evade the immune system [112].

Currently, a majority of the global population has developed immunity to SARS-CoV-2, either naturally as a result of infection or as a result of vaccination. In some cases, this immunity has been achieved through a combination of natural infection and vaccination. This raises the question of whether the presence of antibodies may enhance the risk of infection. In a study conducted by Connir et al., a panel of PVP pseudotyped with S protein variants, including Delta, Beta, and Omicron (BA.1 and BA.2), was used to demonstrate that some NTD-S1 protein-specific antibodies derived from convalescent donors exhibited infection-enhancing activities [113].

## 4. Pseudotyped Virus-Based Assay in Regard to Other RNA Viruses

The development of pseudotyped viruses allowed for the study of a multitude of highly pathogenic and infectious viruses with minimal safety risks.

Pseudotyped viruses that carry the envelope protein of the influenza virus (HA), which is the principal protein for attachment and fusion with the cell, are widely used for the evaluation of continually redesigned vaccines and new developed anti-influenza agents [114].

Prior to the global pandemic caused by SARS-CoV-2, pseudo-viral systems were employed to investigate the cellular tropism of viruses. This resulted in the discovery that SARS-CoV is capable of infecting cells with increased expression of the ACE2 receptor [65], as well as the description of the function of the human foamy virus Bet protein [115]. A comprehensive examination of the function of the human marker for late endosomes/lysosomes, which was shown to serve as a specific receptor for Lassa virus, LAMP1, was conducted by Zhang et al. [116]. Yu et al. demonstrated the impact of a combined substitution in the V3 loop and C4 region of the HIV-1 envelope on co-receptor switching from CCR5 to CXCR4. The impact of these mutations on viral pathogenesis and disease progression was investigated by using two panels of HIV-1-based chimeric pseudoviruses [117].

The use of pseudoviral particles enables the study of the biological characteristics and target cell penetration rates of novel animal viruses with unknown pathogenesis [118,119]. The current focus is on bat SARS-like coronaviruses, which are considered a potential candidate for the next spillover of coronaviruses. The use of pseudotyped viruses allows for the characterization of the interaction of the virus of interest with target cell receptors from different species and its capabilities to infect target cell lines, including human cells [120,121]. Recombinant VSV particles were pseudotyped with a protein derived from the porcine epidemic diarrhoea virus (PEDV) in order to study the process of cellular entry of PEDV and evaluate the potential of viral inhibitors [122].

In order to conduct preclinical trials of pharmacological agents against highly pathogenic viruses such as HIV, Ebola, hepatitis C and others, pseudotyped virus systems were developed [123,124,125,126]. A motif on the hepatitis C virus envelope protein 2 was identified as the agent responsible for suppressing T cell activation [127]. Hu et al. developed a lentiviral pseudovirus-based entry assay utilizing particles bearing the fusion protein of either type A or B respiratory syncytial virus [128]. The pharmacological activity of the promising inhibitors of Lassa virus was evaluated using HIV-1-based pseudotyped viruses [129]. Liang J. et al. employed HIV-1 Env pseudoviruses, generated through co-transfection of a HIV-1 rev/env expression plasmid and an env-deficient HIV-1 backbone plasmid, to investigate and characterize three new trispecific antibodies using a neutralization assay to evaluate their potency against HIV-1 [130]. Luo et al. developed an HIV-based pseudoviral neutralization assay for Nipah virus. The particles were constructed to bear the fusion protein and glycoprotein of the virus of interest [131]. The particles pseudotyped by F or G glycoproteins of Nipah virus, which are involved in the processes of cell attachment and fusion, were employed to validate the role of specific host proteins in the entry process [132].

Furthermore, the detection of nAbs against crucial viral proteins is employed to assess the efficacy of vaccines and to inform the development of post-infection immunity. Xu et al. quantified the levels of polyclonal nAbs present in the serum of individuals who recovered from severe fever with thrombocytopenia syndrome bunya-virus infection using a VSV-based pseudovirus neutralization assay. This local epidemiological study made a significant contribution to the identification of endemic strains and estimation of cross-nAbs levels among the samples [133]. Huttner et al. presented the findings of the study on the long-term clinical efficacy of vaccine rVSV-ZEBOV, a live-attenuated recombinant VSV expressing the Zaire ebolavirus glycoprotein. The series of laboratory tests conducted on the collected serum samples included a VSV-based pseudovirus neutralization assay targeting the Ebola Zaire 95 glycoprotein and the Ebola Zaire Guinea 2014 glycoprotein [134]. In a separate study, Bi et al. demonstrated that three recombinant rabies virus-vectored vaccines encoding glycoproteins of Marburg virus lineages were capable of inducing nAbs in an animal model. The neutralizing activity of mouse sera was measured following vaccination using lentiviral-based pseudoviruses carrying a luciferase reporter gene [135].

## 5. Conclusions and Future Outlook

The integration of PVP technology into this field presents many advantages, including the capacity to examine highly contagious enveloped viruses under biosafety level 2 conditions. The evolution of this technology has enabled the development of a method for producing recombinant viral particles that are deficient in their core and envelope proteins. This technological development enabled researchers to circumvent mutations in the viral particles, ensuring they would not revert to their wild-type state. The directed infection of these particles to specific target cells has also been facilitated, thereby allowing for precise manipulation of the infection process. A notable property of these particles is their capacity for replication, which is limited to a single round. This significantly expands the number of research groups that can participate in such studies globally, as some regions and countries have limited access to BSL-3 and BSL-4 facilities. Consequently, the biology of new emerging viruses can be understood within a relatively short timeframe. It was noteworthy at the outset of the global pandemic caused by the SARS-CoV-2 virus that the scientific community was able to rapidly gain insights into the viral life cycle and its evolution by employing molecular biology, genetics, and PVP technology. The long-term study of viral infections and pandemics provides insights into viral variability, the formation of virus serotypes, and the possibility of different viral variants circulating simultaneously. PVP-based assays have become a valuable tool for assessing population immunity and screening for different viral inhibitors against these viral variants, offering an alternative to live virus-based assays. Moreover, the high-throughput assays are of great importance for the rapid and robust evaluation of immunological parameters among healthy individuals and patients across the globe, as well as a valuable tool for screening viral inhibitors [136].

While the expanded use of the pseudoviral system offers significant benefits to the scientific community, it also presents a number of limitations. PVPs can only be constructed for enveloped viruses, which excludes such epidemiologically significant viruses as rotavirus and poliovirus. Furthermore, the distribution of surface proteins on viruses is not always accurately reflected due to the diverse shapes that viruses can adopt, including spherical, bullet-like, and other forms. Compared to live viruses, PVPs facilitate simulation of the entry process, but do not accurately reflect intracellular processes such as proliferation and the release of newly synthesized viral particles. Furthermore, the calculation of the titers for pseudoviruses remains challenging due to the use of disparate methodologies across different laboratories. For instance, p24 antigen quantification by ELISA or the detection of a specific number of pseudoviral genomes in a defined volume of solution by reverse transcription fluorescence quantitative PCR are two such methods. In the event when a comparison of the results is required, it is recommended to validate the data using the universal standards, namely live virus-based assays [137]. The development of PVP assays for the study of neutralizing immunity against viruses and the screening of potential viral inhibitors has been conducted by different research groups using a variety of protocols and packaging vectors. Several studies have demonstrated that PVP-based assays utilizing different packaging vectors and live virus-based assays exhibit a high degree of correlation with one another [138,139]. However, there is a paucity of studies that directly compare live virus-based and PVP-based assays.

Notwithstanding the aforementioned disadvantages, pseudovirus-based assays are anticipated to emerge as a highly effective and convenient immunological tool with widespread applications in the future.

## Data Availability

This review did not report any new data.
